# Linking mineral chemistry and radiological risk in rare-metal-bearing monzogranite in Egypt

**DOI:** 10.1038/s41598-025-20310-4

**Published:** 2025-11-18

**Authors:** Mohamed M. Ghoneim

**Affiliations:** Nuclear Materials Authority of Egypt, P.O. Box 530, Maadi, 11381 Cairo Egypt

**Keywords:** Monzogranite, Thorite, Hazards, Natural radioactivity, Egypt, Environmental sciences, Nuclear physics

## Abstract

This study aims to define the mineralogical controls on natural radioactivity and its associated hazards in the El Fereyid monzogranite, Egypt. Integrated petrographic, microchemical, and radiometric analyses identify thorite (containing ≤ 66.1 wt% ThO_2_), monazite-(Ce) (≤ 10.0 wt% ThO_2_), and zircon (≤ 1.4 wt% UO_2_) as the primary carriers of radioactivity. These minerals are responsible for ^232^Th and ^238^U activities that significantly exceed global averages. Multivariate statistics confirm strong correlations (r > 0.8) between these Th-bearing phases and elevated gamma dose rates. We conclude that the primary magmatic crystallization and subsequent hydrothermal alteration of these accessory minerals create substantial radiological heterogeneity. Although mean radiological hazard indices remain within international safety limits, localized concentrations generate annual effective doses up to 0.7 mSv and annual gonadal dose equivalents of up to 0.9 mSv, necessitating targeted occupational precautions. This work establishes a direct petrogenetic link between rare-metal enrichment and radioactivity, providing a crucial model for resource potential and radiological risk assessment in analogous granitic terrains worldwide.

**Supplementary Information:**

The online version contains supplementary material available at 10.1038/s41598-025-20310-4.

## Introduction

Naturally occurring radioactive materials (NORMs), primarily uranium (^238^U), thorium (^232^Th), and potassium-40 (^40^K), are heterogeneously distributed within the Earth’s crust. In granitic terrains, their occurrence is predominantly controlled by accessory minerals such as zircon, monazite, and thorite, which act as primary hosts for U and Th. The enrichment of these elements presents a dual significance: economic potential for critical metals and a source of environmental radiological hazard. Understanding the precise mineralogical controls on radioactivity is therefore crucial not only for advancing petrogenetic models but also for developing effective radiation protection strategies in areas with elevated natural background radiation^1,2^.

This study investigates the El Fereyid monzogranite in Egypt’s Eastern Desert, a representative example of rare-metal granitoids within the Arabian-Nubian Shield. We aim to bridge a significant knowledge gap by moving beyond isolated geochemical or radiometric surveys to establish a direct, mechanistic link between specific mineral phases and the observed radiation fields. The primary objectives of this work are to: (1) identify and chemically characterize the key radioactive mineral assemblages using advanced petrographic and microchemical techniques; (2) quantify the activity concentrations of ^238^U, ^232^Th, and ^40^K and calculate associated radiological hazard indices; and (3) employ multivariate statistical analysis to correlate mineral occurrence with radiation anomalies, thereby defining the mineralogical controls on the radiological footprint. By integrating these approaches, this research provides a predictive model for assessing resource potential and environmental safety in analogous shield terrains globally.

## Literature review and background

The Arabian-Nubian Shield (ANS), a vast Precambrian terrane extending across northeastern Africa and western Arabia, hosts numerous rare-metal granitoids known for their enrichment in high-field-strength elements (HFSEs) and rare earth elements (REEs). In Egypt’s Eastern Desert, post-collisional granitoids of the ANS, such as the El Fereyid monzogranite, are particularly noteworthy. These intrusions are characterized by peraluminous, high-silica compositions with elevated potassium and aluminum, and significant enrichment in incompatible elements like thorium and light REEs.These geochemical signatures indicate advanced fractional crystallization, marking them as both potential rare metal resources and natural radiation hotspots^3^.

Previous investigations in the Eastern Desert have often treated economic geology and radiometric assessments as separate disciplines. For instance, studies on the El Fereyid area itself have detailed its geological and geochemical characteristics^3^ and the mineralogy of its associated pegmatites^4^, while geochronological work has constrained its timing^5^. Regional radiometric studies, such as those conducted in the Um Taghir area^6,7^, have provided valuable data on activity concentrations and radiological indices, highlighting that elevated radioactivity is a frequent feature of Eastern Desert granites. Awad et al.^6^ performed a statistical analysis on granite rocks north of Um Taghir, confirming elevated radionuclide activities and calculating associated hazard indices, while their earlier work (2020)^7^ combined geochemical and radiophysical methods to characterize radioactive content.

However, a critical knowledge gap persists: a comprehensive, mineralogically-focused study that directly links the specific mineral hosts (e.g., thorite, monazite, zircon) to the measured radiation fields and hazard indices. Most prior research has reported bulk rock radionuclide activities without detailing the micro-scale mineral chemistry that governs these distributions. This gap limits the ability to predict radiation risks based on petrographic observations and to understand the full petrogenetic history recorded by these radioactive accessories.

The radiogenic potential of these granitoids raises important public health concerns. Chronic exposure to gamma radiation and radon emissions from Th- and U-bearing minerals is epidemiologically linked to increased health risks, necessitating science-based protocols for mining, quarrying, and land use planning^4,8,9^. Therefore, a study that quantitatively characterizes radioactive mineral assemblages and establishes spatial correlations between mineral occurrence and radiation fields is essential.

This work bridges this gap through an integrated investigation of the El Fereyid monzogranite. We combine high-resolution petrography, electron probe microanalysis, in situ gamma spectrometry, and advanced statistical methods. The scientific significance of this approach extends beyond local applications, offering a transferable model for similar assessments in other NORM-rich terrains. The findings will contribute to refining radiation protection guidelines and developing mineralogical proxies for rapid risk evaluation, directly informing sustainable resource development and occupational safety protocols.

## Geological setting

The basement rocks of the Eastern Desert (ED) of Egypt, exposed along the Red Sea margins and Sudan border, predominantly formed during the Neoproterozoic era (900–550 Ma). Geochronological and isotopic studies have revealed that these rocks consist of juvenile Neoproterozoic crust accreted during the Pan-African orogeny and intermixed with reworked older continental fragments^10,11^. The Eastern Desert (ED) is subdivided into three lithostructurally distinct terranes: the Northern, Central, and Southern Eastern Desert (SED)^12^. As part of the Arabian-Nubian Shield (ANS), the SED terrane comprises Neoproterozoic ophiolitic fragments, metavolcanic rocks of island-arc affinity, and subordinate gneisses and metasedimentary schists, all intruded by syn- to late-orogenic granitoids. The post-collisional evolution of the SED terrane was marked by the eruption of the mafic to intermediate Dokhan Volcanics, which are unconformably overlain by molasse-type sedimentary successions^11–13^. Following craton stabilization, numerous granitic intrusions enriched in high field strength elements (HFSEs) (e.g., Zr, Nb, Y, Mo, Ta) and economically significant metals (including Cu, Sn, W, Au, and rare earth elements [REE]) were emplaced within the Arabian-Nubian Shield (ANS) rocks of the Eastern Desert^2,5,14–20^.

The tectonic evolution of the Southern Eastern Desert (SED) was fundamentally controlled by the terminal stages of the East–West Gondwana amalgamation^7^, with five distinct structural episodes recognized. The earliest phase (> 715 Ma) involved NW-WNW-directed folding and thrusting associated with syntectonic tonalite-granodiorite intrusions during arc terrane accretion, forming the Sol Hamed-Onib and Allaqi-Heiani sutures^21^. This was followed by late-orogenic NE–SW compression (580–660 Ma), generating N–S-trending upright folds and the transpressional Hamisana shear zone. Post-orogenic relaxation permitted the emplacement of evolved granitoids (syenogranite-monzogranite suites) and microgranite dikes, followed by ENE–WSW-oriented syncline development. The youngest phase features multi-directional fault systems and dike emplacement, with neotectonic activity persisting through the Phanerozoic, demonstrating prolonged lithospheric memory following the Pan-African cratonization^11^.

**Fig. 1:**
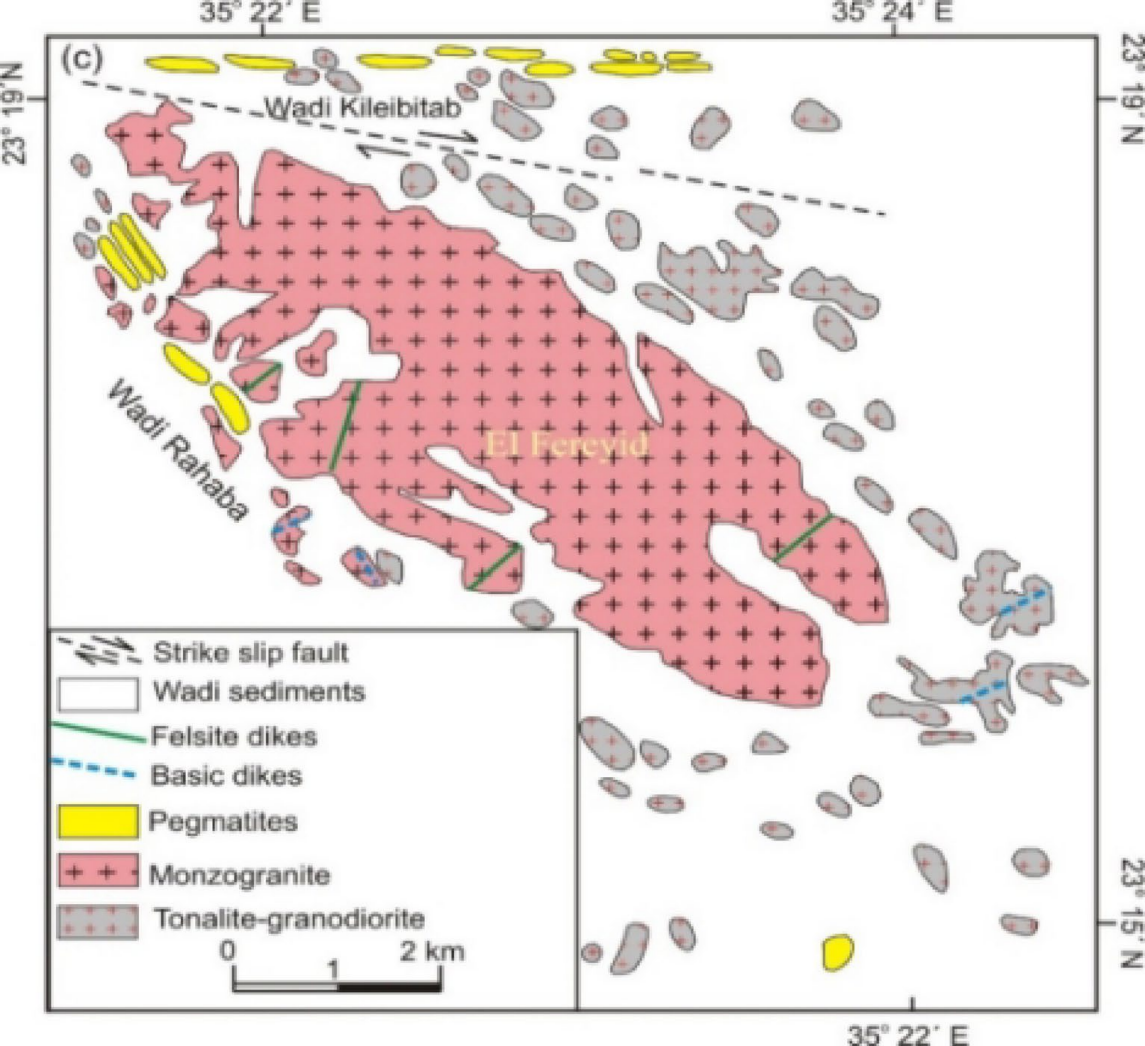
Geologic map of El Fereyid area, Southern part of the Eastern Desert, Egypt Abdel Naby and Saleh, 2003.

Field investigations in the El Fereyid area document a basement complex comprising three principal lithological units: (1) older granitoids (tonalite to granodiorite), (2) younger monzogranite plutons, and (3) late-stage pegmatites crosscut by a declining abundance sequence of mafic to felsic dikes (Fig. 1). The tonalite-granodiorite suite forms scattered, low-to-moderate relief outcrops peripheral to monzongranite bodies, exhibiting gray to whitish-gray coloration, coarse-grained textures, and prominent surficial weathering features including exfoliation joints, fracture networks, and cavernous dissolution structures^3,5,22^.

The tonalite–granodiorite suits are intruded by the monzogranite with sharp intrusive contacts.The U–Pb (SHRIMP–II) age of magmatic crystallization for the El Fereyid monzogranite: 626 ± 13 Ma^5^. The El Fereyid monzogranite is characterized by a calc-alkaline and peraluminous composition, consistent with its classification as an I-type granite^3^.

## Materials and methods

### Analytical methods

#### Petrography and electron probe microanalysis (EPMA)

Twenty thin sections of the studied monzogranite rocks were meticulously prepared and polished to examine their petrographic and mineralogical characteristics. These sections were initially analyzed under a polarizing microscope to identify essential and accessory minerals, as well as secondary alteration products.

The rare metal mineralization within the monzogranite was investigated using electron probe microanalysis (EPMA) with a JEOL JXA-8200 instrument Geomodel Labs at Saint Petersurg State University, Russian Federation following preliminary scanning electron microscopy (SEM) conducted with an EDAX Pegasus 4000 EDS system. Ten polished thin sections were subjected to EPMA under standardized operating conditions, including an accelerating voltage of 15 kV, a beam current of 15 nA, and a beam diameter of 1–2 μm. Measurements were performed using wavelength-dispersive spectrometers (WDS), with peak and background counting times set at 10 and 5 s, respectively.

#### Calibration and quality assurance for EPMA

To ensure analytical accuracy and precision for major, minor, and trace elements, the microprobe was calibrated prior to analysis using a comprehensive suite of natural and synthetic standards. **Natural standards** included: albite (for Na-Kα and Al-Kα), orthoclase (for Si-Kα and K-Kα), hematite (for Fe-Kα), fluorite (for F-Kα), and monazite (for P-Kα, Ca-Kα, and Yb-Lα). **Synthetic reference materials** included: UO_2_ (U-Mβ), ThO_2_ (Th-Mα), ZrO_2_ (Zr-Lα), PbCrO₄ (Pb-Mα), rare earth element (REE) phosphates (Ce-Lα, Nd-Lα, Sm-Lα), GdTiGe (Gd-Lα), DyRu_2_Ge_2_ (Dy-Lα), and YPO₄ (Y-Lα). To monitor and correct for instrumental drift during the extended analysis sessions, a secondary internal standard (e.g., a well-characterized in-house mineral) was measured after every 20 analytical points. The minimum detection limits (MDL) for most elements ranged from 0.01 to 0.05 wt% under these operating conditions. Analytical accuracy, verified by repeated analysis of the standard materials, was better than ± 1–2% for major elements and ± 5–10% for trace elements near detection limits.

#### In-situ gamma-ray spectrometry and dose rate measurement

Terrestrial radionuclide concentrations (U, Th, K) in the studied monzogranite samples were quantitatively determined using an RS-230 BGO gamma-ray spectrometer at **the Nuclear Materials Authority of Egypt.** This portable instrument provides rapid (30–120 s) in situ measurements of uranium and thorium (in ppm) and potassium (in wt%).

#### Calibration and quality assurance for gamma spectrometry

The spectrometer features a factory-loaded, geometry-specific efficiency calibration for the **semi-infinite field geometry**. Its self-stabilizing operation maintains energy calibration, eliminating drift from temperature variations and the need for external calibration sources in the field [23,24]. To ensure data reliability and account for small-scale heterogeneity, **five replicate measurements** were performed at each sampling location. The relative standard deviation (RSD) of these replicates was consistently below 5%, confirming high measurement precision. The measured elemental concentrations were converted to activity concentrations (Bq kg^−1^) using the standard conversion factors endorsed by UNSCEAR^25^: 12.35 Bq kg^−1^ ^238^U per ppm U, 4.060 Bq kg^−1^ ^232^Th per ppm Th, and 313.0 Bq kg^−1^ ^40^K per 1% K. Concurrent with spectral measurements, ambient gamma dose rates were recorded at 1 m above ground level using a calibrated RDS-100 survey meter. The instrument’s calibration is **traceable** to a ^60^Co reference source (7.4 × 10^8^ Bq), with certification provided by the Egyptian National Institute of Standards (ENIST), ensuring the accuracy of the reported dose rates. This integrated approach combining gamma-ray spectrometry with dose rate measurements enables comprehensive radiological characterization of the study area’s granitic bedrock.

The **radium equivalent activity (Raeq)** is a standardized index used to assess the potential radiological health risks posed by naturally occurring radioactive materials (NORMs) such as ^238^U, ^232^Th, and ^40^K. To ensure public safety, international guidelines stipulate that the annual effective dose (AED) for the general population should not exceed 1 mSv per year, corresponding to a Raeq limit of 370 Bq/kg^26^.

The **Raeq** is calculated as follows:

1 $${\text{Raeq}}\;\left( {{\text{Bq}}\;{\text{kg}}^{{ - {1}}} } \right) = {\text{ AU }} + { 1}.{\text{43 ATh }} + \, 0.0{\text{77 AK}}$$

where A_U_​, A_Th_​, and A_K_​ are the activity concentrations (Bq kg^−1^) of ^**238**^**U, **^**232**^**Th, and **^**40**^**K**, respectively.The coefficients **1.43** and **0.077** account for the relative gamma dose contributions of ^**232**^**Th** and ^**4**0^**K** compared to ^**238**^**U**^**27**^.

### Calculation of absorbed dose rate and annual effective dose

The **absorbed dose rate in air (D**_**air**_**)** at 1 m above the ground, primarily due to gamma radiation, is given by:

2 $${\text{D}}air\left( {{\text{nGy}}\;{\text{h}}^{{ - {1}}} } \right) \, = \, 0.{43}0{\text{ A}}_{{\text{U}}} + \, 0.{\text{666 A}}_{{{\text{Th}}}} + \, 0.0{\text{42 A}}_{{\text{K}}}$$

The **annual effective dose (AED)** received by individuals is determined for both **outdoor (AEDout)** and **indoor (AEDin)** exposures, accounting for occupancy factors (20% outdoor, 80% indoor)^28,29^.

3 $${\text{AED}}_{{{\text{out}}}} \left( {{\text{mSv}}/{\text{y}}} \right) \, = {\text{ D}}_{{{\text{air}}}} \left( {{\text{nGy}}/{\text{h}}} \right) \, \times \, 0.{2} \times { 876}0 \, \left( {{\text{h}}/{\text{y}}} \right) \, \times \, 0.{7 }\left( {{\text{Sv}}/{\text{Gy}}} \right) \, \times { 1}0^{{ - {6}}} \left( {{\text{mSv}}/{\text{nGy}}} \right)$$

4 $${\text{AED}}_{{{\text{in}}}} \left( {{\text{mSv}}/{\text{y}}} \right) \, = {\text{ D}}_{{{\text{air}}}} \left( {{\text{nGy}}/{\text{h}}} \right) \, \times \, 0.{8} \times { 876}0 \, \left( {{\text{h}}/{\text{y}}} \right) \, \times \, 0.{7 }\left( {{\text{Sv}}/{\text{Gy}}} \right) \, \times { 1}0^{{ - {6}}} \left( {{\text{mSv}}/{\text{nGy}}} \right)$$

where: **8760 h/y** = total hours in a year. **0.7 Sv/Gy** = dose conversion coefficient from absorbed dose to effective dose. **10**^**−6**^** mSv/nGy** = unit conversion factor.

**Excess Lifetime Cancer Risk (ELCR)** estimates the probability of an individual developing fatal cancer due to prolonged radiation exposure and is computed as:

5 $${\text{ELCR }} = {\text{ AED }} \times {\text{ DL }} \times {\text{ RF}}$$

where: **AED** = total annual effective dose (sum of indoor and outdoor exposures). **DL** = average life duration (70 years, as per ICRP 103). **RF** = risk factor (0.05 Sv^−1^ for fatal cancer risk).

The radiological hazard assessment employed also indices: the external hazard index (H_ex_) to evaluate gamma radiation exposure risk from ^238^U, ^232^Th, and ^40^K, and the internal hazard index (H_in_) to assess inhalation risks from radon and its progeny, where A_U_, A_Th_, and A_K_ represent the activity concentrations (Bq kg^−1^) of each radionuclide^15^. The denominator values correspond to the maximum permissible concentrations established by UNSCEAR (2000)^25^ with H values exceeding unity indicating potential radiological concern. These indices provide comprehensive evaluation of both external and internal radiation exposure pathways in accordance with current international radiation protection standards^28,29^. calculated as

6 $${\text{H}}_{{{\text{ex}}}} \, = \, \left( {{\text{A}}_{{\text{U}}} /{37}0} \right) \, + \, \left( {{\text{A}}_{{\text{K}}} /{481}0} \right) + \, \left( {{\text{A}}_{{\_{\text{Th}}}} /{259}} \right)$$

7 $${\text{H}}_{{{\text{in}}}} \, = \, \left( {{\text{A}}_{{\text{U}}} /{185}} \right) \, + \, \left( {{\text{A}}_{{{\text{Th}}}} /{259}} \right) \, + \, \left( {{\text{A}}_{{\text{K}}} /{481}0} \right)$$

The Annual Gonadal Equivalent Dose (AGED) was determined to evaluate the radiological risk to reproductive organs, using the well-established formula from UNSCEAR (2000):

8 $${\text{AGED }}\left( {{\text{mSv}}/{\text{year}}} \right) = \left( {{3}.0{\text{9A}}_{{{\text{Ra}}}} + { 4}.{\text{19A}}_{{{\text{Th}}}} + \, 0.{\text{314A}}_{{\text{K}}} } \right)/{ 1}000$$

where A_Ra_, A_Th_, and AK are the activity concentrations (in Bq kg^−1^) of ^226^Ra, ^232^Th, and ^40^K, respectively. The resulting values were converted to millisieverts per year (mSv y^−1^)^30^.

## Results and discussion

### Petrographic description

**Fig. 2:**
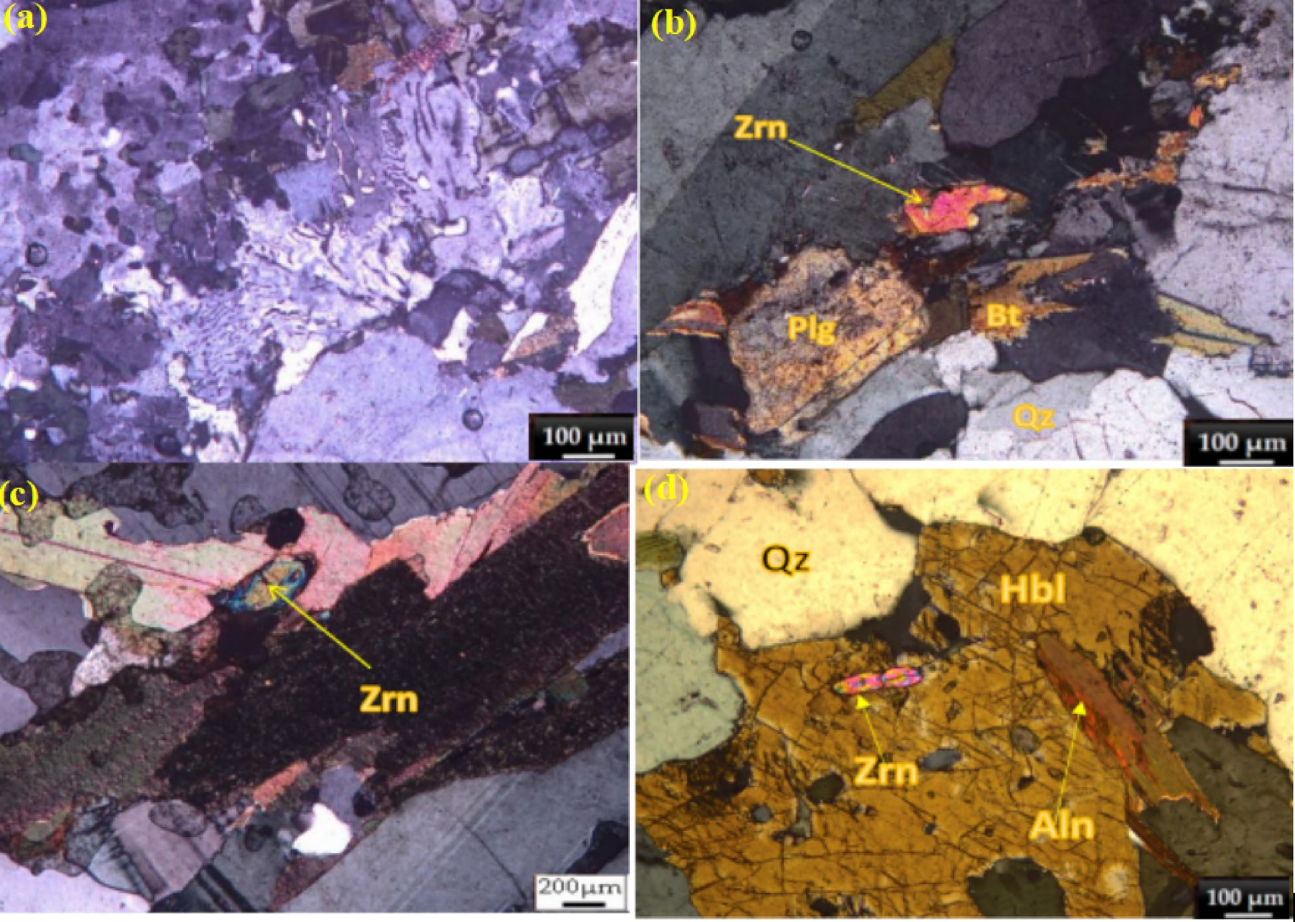

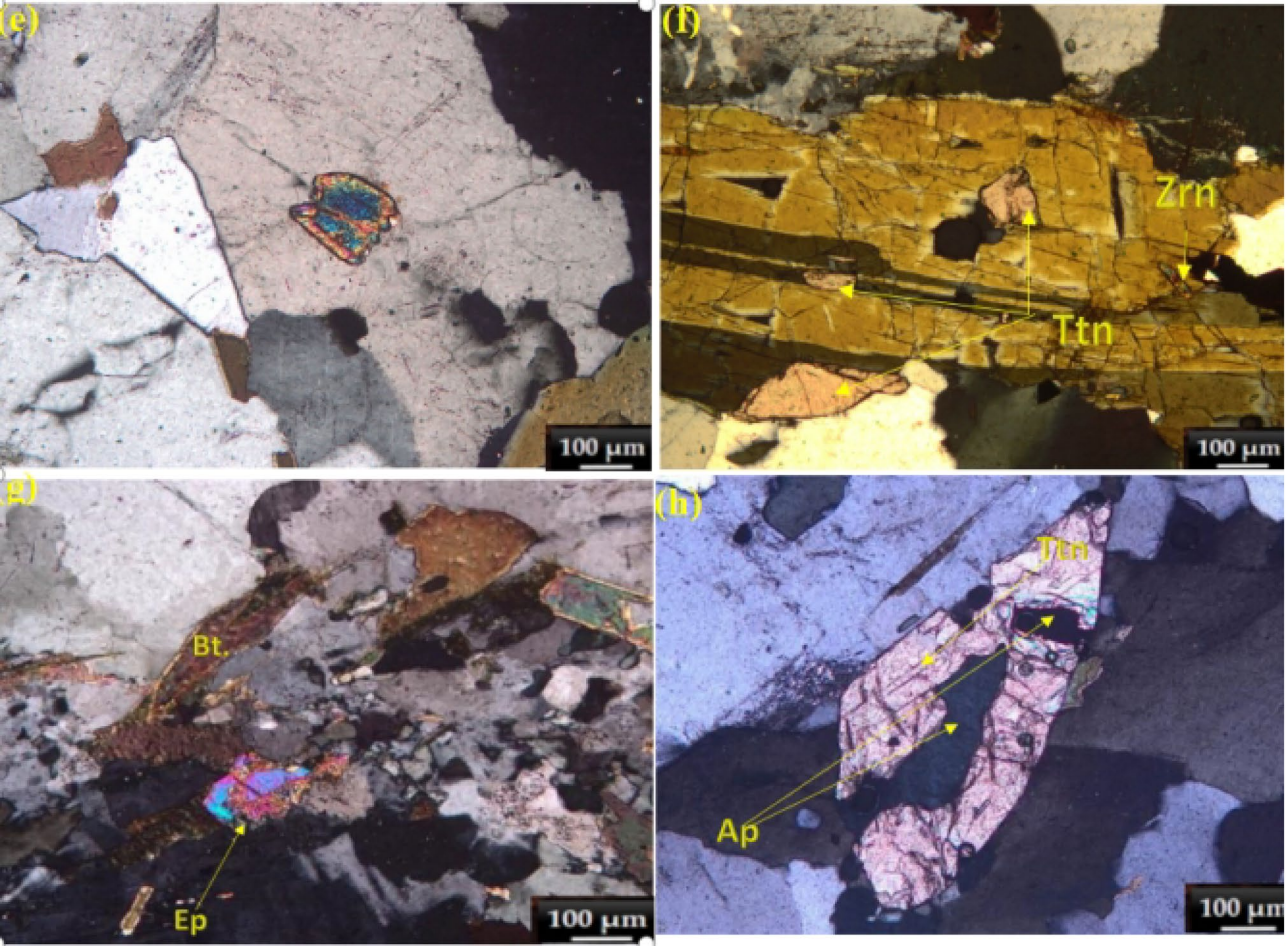
Photomicrographs illustrating textural and mineralogical features of the monzogranite from the El Fereiyd area, Southeastern Desert (SED) of Egypt. (**a**) Myrmekitic texture; (**b**) Zircon (Zrn) associated with plagioclase (Plg) and quartz (Qz); (**c**) Zircon crystal in association with biotite (Bt); (**d**) Chain-like arrangement of zircon crystals associated with allanite (Aln) inclusions within hornblende (Hbl). Photomicrographs of the monzogranite from the El Fereiyd area, Southeastern Desert (SED) of Egypt. (**e**) Parallel twinning in zircon crystals within a quartz matrix; (**f**) Titanite (Ttn) crystals associated with zircon, embedded in hornblende; (**g**) Epidote (Ep) associated with biotite (Bt) and quartz (Qz); (**h**) Association of apatite (Ap), titanite (Ttn), and quartz (Qz).

**Fig. 3:**
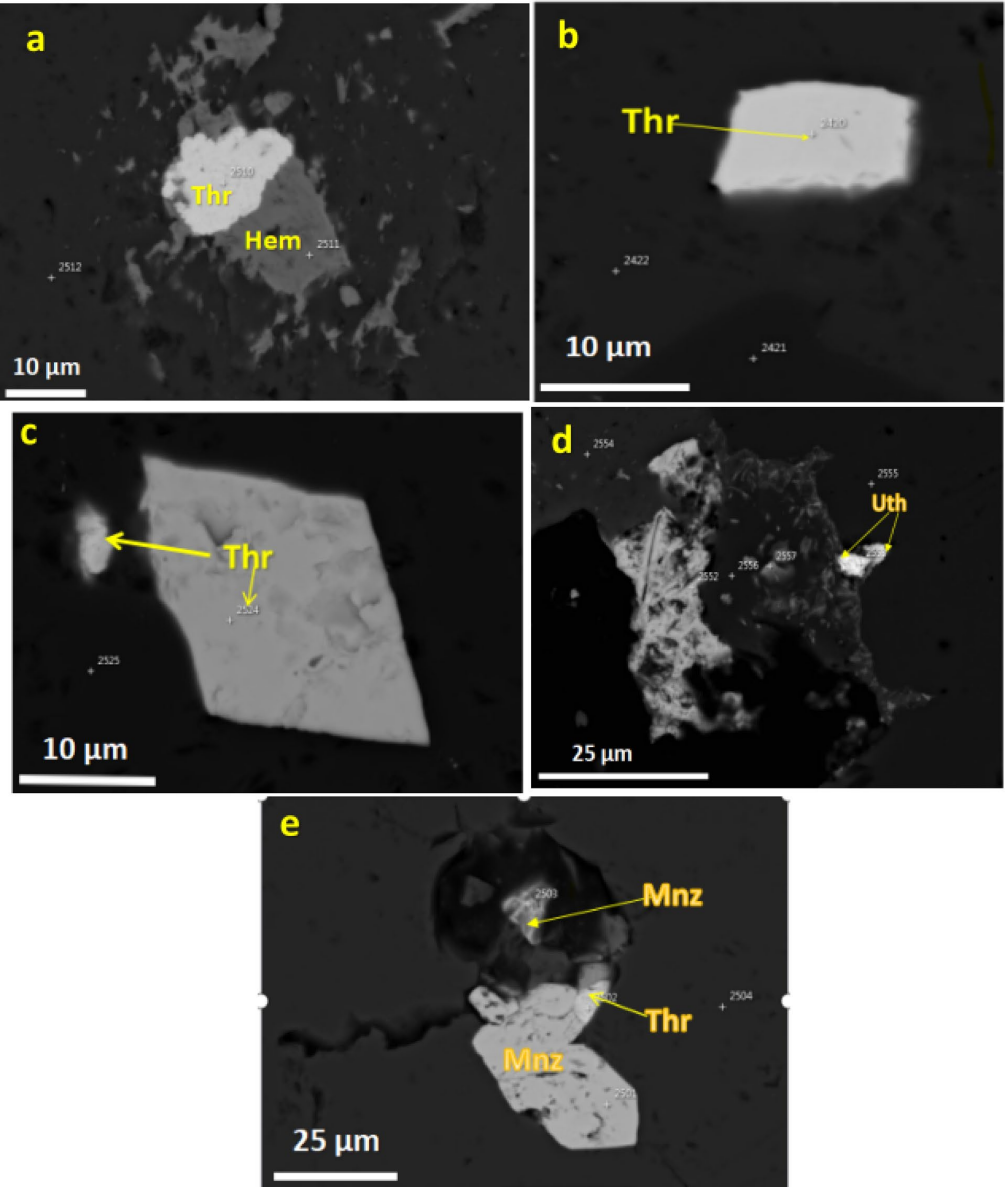
Back-scattered images of Th-,U-, REE-bearing mineralization from monzoranite at the study area, South Eastern Desert, Egypt. (**a**) Association of thorite and hematite of irregular, patchy intergrowth pattern against the dark background matrix of plagioclase; (**b**) Homogeneous thorite crystal of elongated rectangle with smoothed corners; (**c**) Irregular, polygonal shape with somewhat angular edges; (**d**) Fine-grained uranothorite; (**e**) Two distinct bright masses of monazite associated with fine grained thorite. *Abbreviations* Thr, thorite; Hem, hematite;Uth, uranothorite; Mnz, monazite.

Microscopically, the monzogranite exhibits a reddish-pink to red coloration and is characterized by a coarse-grained texture, displaying both porphyritic and hypidiomorphic-equigranular features. The primary mineral constituents of the rock include plagioclase, quartz, K-feldspar, biotite, and hornblende. Accessory mineral phases identified comprise zircon, titanite, apatite, and opaque minerals such as magnetite (Fig. 2a–h). Plagioclase occurs as anhedral to subhedral prismatic crystals, exhibiting a cloudy morphology due to extensive and/or partial alteration to sericite. The anorthite content is estimated to range between 25–30%. Quartz is present as anhedral to subhedral crystals, predominantly filling interstitial spaces between other mineral components. It displays undulose extinction, indicative of strain deformation, and is occasionally associated with myrmekitic textures (Fig. 2a). K-feldspar is observed as anhedral to subhedral crystals, primarily orthoclase, with perthitic textures. Some crystals are partially altered to sericite or coated with a dusty kaolinite layer. Biotite is the most abundant mafic mineral, occurring as anhedral to subhedral crystals. It exhibits strong pleochroism, ranging from pale green to dark brown. Biotite is altered to chlorite, with iron oxy-hydroxides developing along cleavage planes. Epidote is sporadically associated with biotite and quartz (Fig. 2g). Zircon is frequently encapsulated within biotite and quartz, displaying zonation and parallel twinning in certain instances (Fig. 3b,c,e). Hornblende is present as anhedral to subhedral crystals, displaying simple twinning. It is frequently altered to chlorite and poikilitically encloses quartz and opaque minerals. Irregular hornblende crystals occasionally contain zircon and apatite as inclusions (Fig. 2d,f). Chain-like arrangements of zircon crystals associated with allanite inclusions are also observed within hornblende (Fig. 3d). Alteration features, such as the sericitization of feldspar and chloritization of biotite and hornblende, are widespread throughout the sample. Zircon occurs as prismatic crystals with high relief, showing zonation. It is frequently enclosed within biotite and quartz and exhibits parallel twinning in some instances (Fig. 2b,c,e). Zircon is also associated with plagioclase and quartz (Fig. 2b) and forms chain-like arrangements with allanite inclusions in hornblende (Fig. 2d).Titanite is found in association with quartz, biotite, and apatite, occurring as subhedral to anhedral crystals (Fig. 3f,h). It is also embedded in hornblende alongside zircon (Fig. 2f). Apatite is present as small rod-like crystals, often enclosed within hornblende and associated with titanite (Fig. 2h). It is also found in association with quartz and titanite (Fig. 2h).

### Radioactive minerals and radioactive bearing minerals

**Fig. 4:**
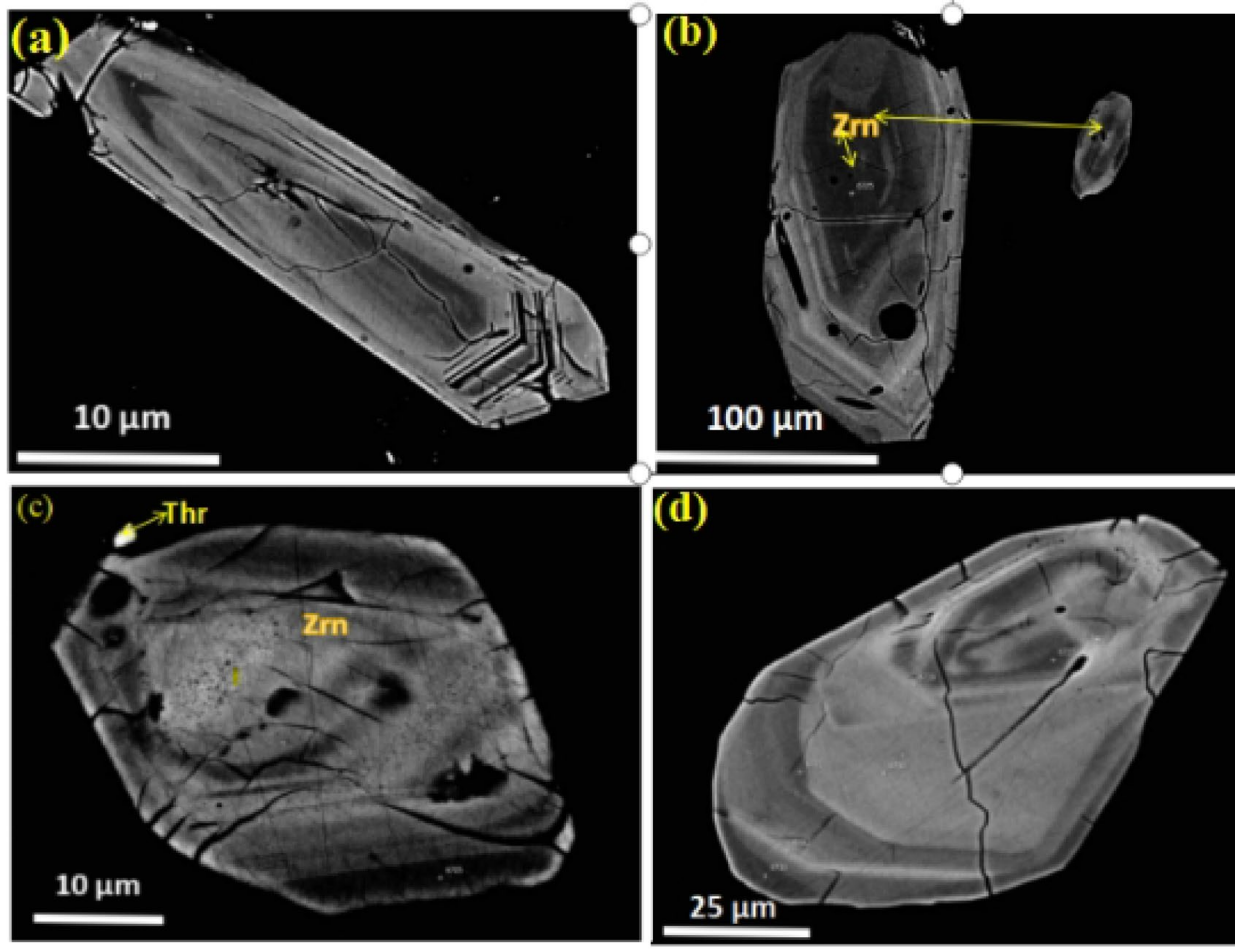
(**a**) An elongated prismatic zircon crystal with sharp edges and distinct growth zones visible within its structure; (**b**) Subhedral zircon crystals exhibit partial rounding and fracture networks; (**c**) rectangular zircon crystal with internal textures and associated with visible thorite inclusion; (**d**) Irregular rounded grains with zonation and cracks inside.

**Thorite** occurs as subhedral to anhedral crystals measuring 10–25 μm in size, typically associated with zircon, hematite, and monazite (Fig. 3). Back-scattered electron imaging reveals textural relationships between thorite and associated minerals in the monzogranite samples from the South Eastern Desert, Egypt. The images document: (1) irregular, patchy intergrowths of thorite and hematite within plagioclase matrix (Fig. 3 a); (2) homogeneous thorite crystals with elongated rectangular morphology and smoothed corners (Fig. 3b); (3) irregular polygonal forms with angular edges (Fig. 3c); (4) fine-grained uranothorite occurrences (Fig. 3d); and (5) two distinct monazite masses associated with fine-grained thorite (Fig. 3e). The back scattered electron contrast in thorite varies significantly due to substitution of Th^4+^ by U^4+^ and REE^3+^, resulting in brighter domains in U/REE-enriched zones^31^. Textural evidence suggests complex alteration relationships, where hematite may replace thorite along fractures during retrograde metamorphism, or conversely, thorite may form through infiltration of Th-bearing solutions into Fe-rich lithologies.The chemical compositions of representative thorite crystals are provided in Supplementary Table S1. Electron microprobe analyses demonstrate that thorite is dominated by ThO_2_ (55.3–66.1 wt%) with significant SiO_2_ (15.9–23.2 wt%) and variable UO_2_ (1.89–6.89 wt%) contents. The mineral exhibits notable concentrations of rare earth elements, including La_2_O_3_ (0.05–1 wt%), Ce_2_O_3_ (0.3–1.5 wt%), Nd_2_O_3_ (0.1–1.5 wt%), Sm_2_O_3_ (0.5–2.3 wt%), Gd_2_O_3_ (0.8–2 wt%), Dy_2_O_3_ (0.02–1.5 wt%), Er_2_O_3_ (0.05–1 wt%), and Y_2_O_3_ (0.5–2.5 wt%). Minor components comprise P_2_O₅ (0.4–1.73 wt%), CaO (0.2–0.93 wt%), and Fe_2_O_3_ (1.42–3.38 wt%). Normalized elemental ratios (Si = 1) show considerable variation in Th (0.706–0.847) and U (0.024–0.08) abundances.

**Uranothorite** occurs as very fine scattered irregular crystals associated with monazite and zircon. The chemical compositions of representative uranothorite crystals are provided in Supplementary Table S2. The analytical data demonstrate an inverse relationship between ThO_2_ (47.6–61.8 wt%) and UO_2_ (5.4–18.3 wt%) contents.The SiO_2_ content (12.5–17.5 wt%) (P_2_O₅ 0.7–2.8 wt%). The rare earth element (REE) content (ΣREE_2_O_3_ 3.2–7.5 wt%) demonstrates enrichment in light REEs (Ce > La > Nd) over heavy REEs (Y), consistent with the typical REE distribution patterns in thorium-bearing minerals. The structural formulae, calculated on the basis of four oxygen atoms, (Th_0.62–0.78_U_0.08–0.24_REE_0.15–0.27_). The increasing uranium content correlates with higher REE incorporation, suggesting charge compensation mechanisms where REE^3+^ substitution accompanies U^4+^ incorporation. Minor elements show distinct distribution patterns:Fe_2_O_3_ (0.9–3.0 wt%) appears enriched in uranium-rich samples, possibly indicating secondary alteration or coupled substitution with REEs. CaO (0.5–2.3 wt%) shows higher concentrations in samples with elevated uranium and phosphorus contents. PbO (0.2–0.9 wt%) likely represents radiogenic lead from uranium and thorium decay. The geochemical variations among the analyzed uranothorite samples reflect multiple substitution mechanisms operating in the crystal structure:Primary substitution: Th^4+^ ↔ U^4+^ (dominant in all samples); Coupled substitutions: (Th^4+^ + Si^4+^) ↔ (U^4+^ + P^5+^) and (Th^4+^) ↔ (REE^3+^ + Na^+^/Ca^2+^); Secondary alteration: evidenced by Fe enrichment in uranium-rich varieties. These compositional variations suggest that the uranothorite formed under changing physicochemical conditions, with later-stage fluids introducing uranium, phosphorus, and rare earth elements into the mineral structure^31^.Late-stage hydrothermal fluids significantly modified U/Th distribution, as evidenced by:1.Thorite alteration to hematite (Fig. 3a): Fracture-controlled Fe-oxide replacement textures suggest fluid-driven Th release, potentially increasing radionuclide mobility in weathering environments.2.Uranothorite micro-veining (Fig. 3e): Secondary U enrichment along fractures implies fluid-assisted U transport, consistent with higher U/Th ratios in altered zones (Supplementary Table S2). 3.Radial cracks in zircon (Fig. 4b): Metamictization-enhanced fluid infiltration likely mobilized trace U, as reflected in heterogeneous UO_2_ concentrations (0.06–1.4 wt%).These secondary processes directly impact radiological hazards: •Enhanced radon emanation potential from fracture networks in altered thorite. •Variable leaching risks—hematite-associated Th may be less mobile than U in uranothorite veins^32^.

**Monazite** in the El Fereyid monzogranite typically occurs as subhedral to anhedral crystals, exhibiting a grain size of approximately 15–20 μm. These phosphate minerals show a clear textural affinity for felsic mineral phases, being predominantly hosted within K-feldspar, albite, and quartz matrices^32^. Monazite frequently forms intimate associations with zircon and thorite, suggesting their co-crystallization from late-stage residual melts^33^. The consistent association with thorite and zircon, particularly in samples exhibiting elevated thorium concentrations, implies these accessory minerals collectively represent significant repositories of both rare earth elements and radioactive constituents in the monzogranite system^34^. The frequent occurrence of monazite in association with thorite and zircon suggests these minerals may have co-precipitated from melts enriched in both rare earth elements and high field strength elements^35^. The chemical composition of the analyzed monazite grains are shown in Table S3 in the supplementary materials.

The monazite crystals from the El Fereyid monzogranite exhibit 29.2–31.5 wt% P_2_O₅ and show significant enrichment in light rare earth elements (LREEs), with total REE oxide (ΣREE_2_O_3_) contents ranging from 58.4 to 68.6 wt%. The REE distribution follows a typical pattern for magmatic monazite, with Ce_2_O_3_ (24.9–30.2 wt%) being the dominant oxide, followed by Nd_2_O_3_ (12.5–14.5 wt%), La_2_O_3_ (10.0–12.5 wt%), Pr_2_O_3_ (3.0–3.9 wt%), and Sm_2_O_3_ (3.5–4.4 wt%). Heavy rare earth elements are present in minor amounts, limited to Gd_2_O_3_ (2.1–3.3 wt%) and Y_2_O_3_ (0.9–1.5 wt%), consistent with the preferential incorporation of LREEs into the monazite structure due to ionic radius considerations^35,36^. The radioactive element content shows moderate concentrations of thorium (6.2–10.0 wt% ThO_2_) and lower uranium levels (0.38–0.48 wt% UO_2_), yielding Th/U ratios of 13–26 that are characteristic of magmatic monazite in reduced granitoid systems^37^. Silicon occurs as a minor component (1.0–2.0 wt% SiO_2_), suggesting limited substitution via the huttonite exchange vector (Th^4+^ + Si^4+^ ↔ REE^3+^ + P^5+^).

The average structural formula, calculated on the basis of 4 oxygen atoms, is (Ce_0.39_La_0.16_Nd_0.18_Pr_0.05_Sm_0.05_Gd_0.04_Y_0.03_Th_0.06_U_0.004_)apfu (P_1.00_Si_0.06_)apfu O₄ confirming the monazite-(Ce) classification^38^. The pronounced Ce-dominance (0.39 apfu) over other REEs reflects the characteristic fractionation pattern of LREEs in evolved granitic melts^39^. The consistently low U/Th ratios (0.03–0.06) are typical of monazite crystallized from reduced, calc-alkaline magmas^40^. These compositional features have important implications for both the petrogenetic history of the host monzogranite and the assessment of its radiological properties, as the monazite represents a significant reservoir of both rare earth elements and radioactive thorium^34^.

**Zircon** crystals exhibit diverse morphologies, reflecting complex crystallization histories and post-formational alteration. Euhedral grains display well-developed prismatic habits with distinct oscillatory zoning (Fig. 4a), indicative of magmatic growth under fluctuating physicochemical conditions^41^. Subhedral specimens exhibit partial rounding and fracture networks (Fig. 4b-c), likely resulting from transport-related abrasion or metamictization-induced volume expansion^42^. Rounded grains with subdued zoning (Fig. 4d) suggest prolonged sedimentary reworking or chemical dissolution^43^. Ubiquitous fracturing across all morphological types points to post-crystallization brittle deformation, while mineral inclusions (Fig. 4b) preserve evidence of coeval melt/fluid entrapment. Grain sizes range from 10 to 200 µm, indicating moderate dimensional variability. Many crystals exhibit corrosion features, including dark patches and fractures, consistent with alteration or metamictization. Back-scattered electron (BSE) imaging reveals bright rim contrasts, suggesting compositional zoning or enrichment of higher atomic number elements along grain margins. Weak zoning patterns, characterized by darker inherited magmatic cores surrounded by oscillatory-zoned rims, further attest to multistage growth. The pervasive fracturing likely records external stresses during metamorphism, underscoring a complex paragenesis involving magmatic crystallization, metamorphic overprinting, and potential fluid-mediated alteration.The chemical composition of representative analyzed zircon grains are listed in the Supplementary Table S4. Zircon grains exhibits significant variability in major and trace elements, reflecting diverse crystallization and alteration conditions. SiO_2_ content ranges from 29.6 to 34.4 wt%, while ZrO_2_ varies between 57.9 and 65.6 wt%, consistent with zircon’s ideal stoichiometry (ZrSiO₄) but indicating minor substitutions and impurities. HfO_2_ (0.1–4.5 wt%) and trace elements such as UO_2_ (0.06–1.4 wt%), ThO_2_ (0.3–1.9 wt%), and Y_2_O_3_ (0.02–1.4 wt%) show notable fluctuations, suggesting variations in melt composition or post-crystallization metasomatism. Elevated Hf, U, and Th concentrations in some grains imply crystallization from evolved magmas or late-stage hydrothermal enrichment. The presence of Fe_2_O_3_ (up to 1.02 wt%) and CaO (≤ 0.4 wt%) may indicate secondary alteration or inclusions of accessory phases. Cation calculations (per formula unit) reveal Si (0.4–0.54 apfu) and Zr (0.46–0.53 apfu) deviations from ideal values (Si = 1, Zr = 1), likely due to radiation-induced metamictization or coupled substitutions involving REE^3+^ + P^5+^ ↔ Zr^4+^ + Si^4+^.

Textural analysis reveals a dual paragenesis for the radioactive mineral assemblage, involving both primary magmatic crystallization and subsequent hydrothermal reworking.

Primary magmatic origins are evidenced by euhedral thorite and zircon crystals displaying intact oscillatory zoning (Fig. 4a). Their occurrence as pristine inclusions within unaltered magmatic phases such as biotite and quartz (Fig. 2b,c) further confirms their early, syngenetic formation. Similarly, the uniform distribution of monazite-(Ce) within feldspar and quartz matrices (Fig. 3e) is consistent with direct crystallization from a late-stage residual melt.

Secondary hydrothermal origins are indicated by textural modifications post-dating magmatic crystallization. These include the occurrence of fracture-filling thorite associated with hematite alteration halos, suggesting fluid-induced remobilization (Fig. 3a). Furthermore, patchy and irregular Th-U zoning patterns within zircon (Fig. 4b) and intricate thorite-monazite intergrowths along grain boundaries (Fig. 3e) are characteristic of fluid-mediated dissolution-reprecipitation and metasomatic replacement processes.

### Assessment of radiological hazards using radium equivalent activity (Raeq) and associated parameters

#### Distribution and variability of radionuclides

**Fig. 5:**
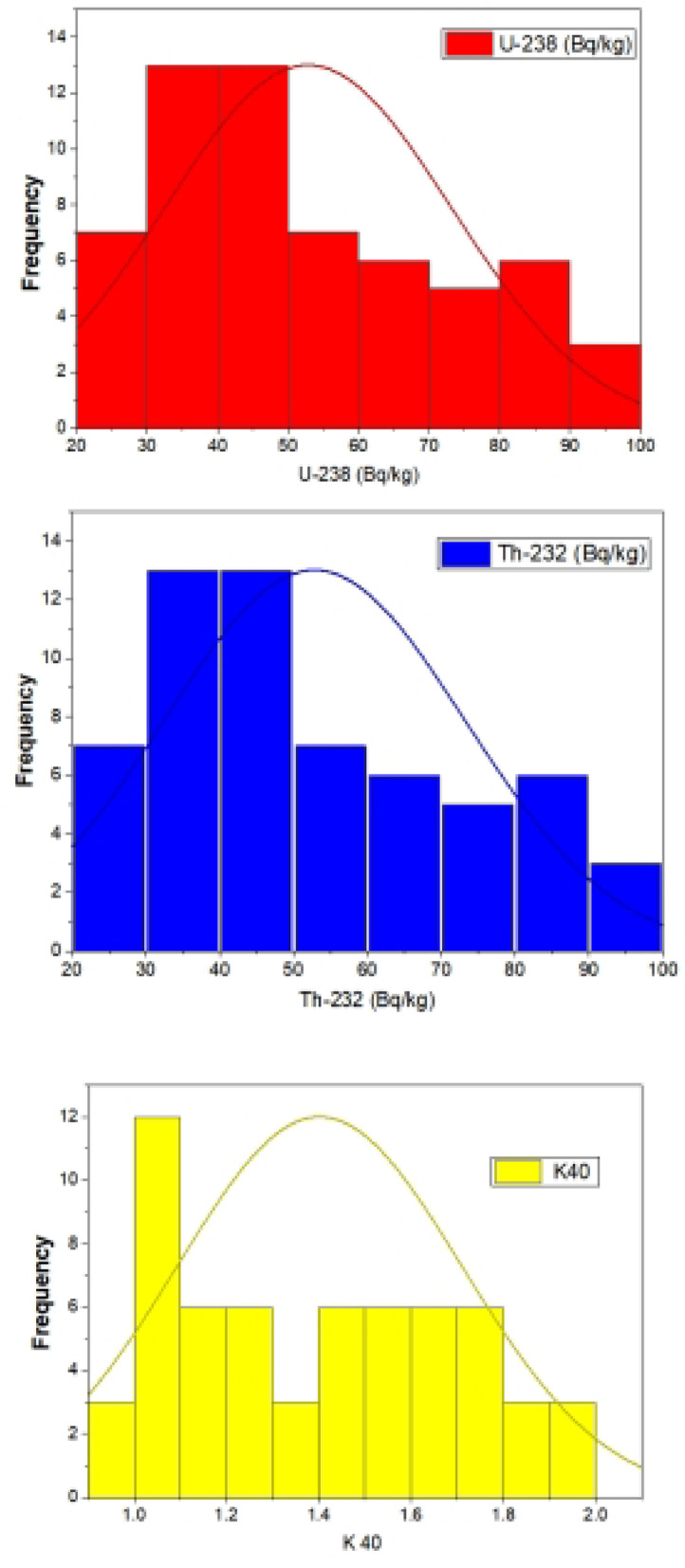
The frequency distributions of ^238^U, ^232^Th, and ^40^K in El Fereyid monzogranite.

Table S5 (Supplementary Materials) presents the measured and calculated radiological parameters for the examined monzogranite samples, while Table 1 summarizes the statistical distribution of radiological hazard indices. Statistical analysis of radioactivity concentrations for ^232^Th, ^238^U, and ^40^K in environmental samples (N = 60) reveals distinct variability and distribution patterns. ^232^Th exhibited the highest mean concentration (76.2 Bq/kg) and variability (coefficient of variation, CV = 44.7%), followed by ^238^U (49.9 Bq/kg, CV = 39.8%) and ^40^K (1.4%, CV = 21.9%). Figure 5 shows the frequency distribution of ^238^U, ^232^Th, and ^40^ K in El Fereyid monzogranite. The distributions of ^232^Th and ^238^U displayed positive skewness (0.12 and 0.45, respectively), indicating a tendency toward higher values, whereas ^40^K exhibited near-symmetry (skewness = 0.08). All radionuclides showed negative kurtosis, suggesting flatter distributions compared to a normal distribution. Concentration ranges varied significantly: ^232^Th (20.7–140 Bq/kg), ^238^U (23.5–96 Bq/kg), and ^40^K (0.9–1.9%). The results align with the observations of (Abdel Gawad et al., 2021)^44^, who reported that ^232^Th concentrations in Wadi sediments are significantly higher than those of ^238^U. This disparity can be attributed to thorium’s lower susceptibility to leaching and greater resistance to weathering compared to uranium, leading to preferential enrichment of thorium-bearing minerals in sedimentary and granitic rocks^44,45^. The consistently higher ^40^K activity concentrations can be explained by the presence of potassium-rich minerals, such as feldspars, micas, and clay minerals^44^.

**Table 1:** Summary statistics of measured radionuclides (Bq/kg) and derived radiological parameters in El Fereyid monzongranite.

Parameter	Mean	Min	Max	Std. Dev	CV (%)	Skewness	Kurtosis
^232^Th (Bq/kg)	76.23	20.7	139.8	34.12	44.7	0.12	− 1.08
^238^U (Bq/kg)	50	23.5	96.3	19.8	39.8	0.45	− 0.92
^40^ K (%)	1.4	0.9	1.99	0.31	21.9	0.08	− 0.54
^226^Ra(Bq/kg)	45	6.9	88.1	25.18	56.3	0.87	− 0.76
Dose Rate (nGy/h)	70.5	28.2	112.3	16.72	23.7	0.31	− 0.38
Outdoor (mSv/y)	0.08	0.03	0.1	0.02	23.7	0.31	− 0.38
Indoor (mSv/y)	0.3	0.1	0.5	0.08	23.7	0.31	− 0.38
Total Dose (mSv/y)	0.4	0.2	0.7	0.1	23.7	0.31	− 0.38
Ra_e_q (Bq/kg)	157	68	258	40.3	25.7	0.25	− 0.45
H_ex_	0.4	0.2	0.7	0.1	25.7	0.25	− 0.45
H_in_	0.57	0.2	0.95	0.15	25.7	0.25	− 0.45
I_γ_	1.09	0.45	1.72	0.29	26.6	0.23	− 0.42
ELCR (× 10^−3^)	1.52	0.6	2.4	0.38	25	0.27	− 0.48
AGED (mSv/y)	0.6	0.2	0.9	0.2	26.6	− 0.15	− 0.33

The mean ^232^Th/^238^Uratio (~ 1.5) observed in the El Fereyid monzogranite is significantly lower than the global average for granitic rocks (~ 3–4)^46^. This deviation may be attributed to several interrelated processes. First, uranium appears preferentially retained within zircon crystals (up to 1.4 wt% UO_2_; Supplementary Table S4), which exhibit high resistance to alteration, whereas thorium is predominantly incorporated into more alteration-prone mineral phases such as thorite and monazite. Second, hydrothermal fluids acting during late-stage magmatic processes likely facilitated greater mobility and remobilization of uranium relative to thorium, as indicated by the presence of uranothorite micro-veins (Fig. 3e) and hematite-thorite alteration textures (Fig. 3a). Third, the low Th/U ratio is consistent with the reduced redox state characteristic of the ilmenite-series monzogranite, where tetravalent uranium (U^4+^) is stabilized within zircon crystals compared to hexavalent uranium (U^6+^), which is more mobile in oxidative fluid environments^47^. Collectively, these factors provide a coherent explanation for the distinctive Th/U geochemical signature observed and its implications for the mobility of radionuclides in analogous granitic systems.

#### Radiological hazards

The radiological hazard assessment of the provided dataset reveals significant variability in radiation exposure and associated risks across the samples. The dose rates range from 28.2 to 112.3 nGy/h, with corresponding outdoor and indoor annual effective doses ranging from 0.03to 0.138 mSv/year and 0.1 to 0.5 mSv/year, respectively. The total annual effective dose attributable to gamma radiation from the El Fereyid monzogranite ranges from 0.2 to 0.7 mSv/year (Table 1). This represents the additive dose from outdoor and indoor exposures, calculated based on the measured activity concentrations of ^232^Th, ^238^U, and ^40^K. This gamma dose component constitutes a significant fraction (up to ~ 29%) of the total global average annual background dose of 2.4 mSv/year (UNSCEAR, 2000)^25^, which includes contributions from inhaled radon, internal emitters, and cosmic rays. The elevated end of this range (0.7 mSv/y) indicates that the monzogranite bedrock is a substantial source of localized terrestrial radiation. While the mean values remain within safety limits, this heterogeneity creates areas where occupational exposure for prolonged periods would require monitoring to ensure adherence to the ALARA (As Low As Reasonably Achievable) principle. The radium equivalent activity (Ra_e_q) ranges from 68 to 258 Bq/kg. All values are below the recommended safety limit of 370 Bq/kg for building materials, indicating a low radiological hazard from external gamma radiation in the studied area^28^.The external hazard index (Ex) and internal hazard index (In) range from 0.184 to 0.6 and 0.2 to 0.9 , respectively. While most values remain below the safety threshold of 1, indicating minimal risk, some samples approach or exceed this limit, particularly those with higher Ra_e_q values. The radioactive level index (Iɣ) ranges from 0.4 to 1.7, with values above 1 indicating elevated radiation levels that may pose health risks. Excess lifetime cancer risk (ELCR) varies between 0.0006 and 0.0024, with higher values associated with increased long-term exposure risks. The annual gonadal equivalent dose (AGED) ranges from 0.2 to 0.9 mSv/year, further emphasizing the variability in radiation exposure across the samples. The annual gonadal equivalent dose (AGED) ranges from 0.2 to 0.9 mSv/year, further emphasizing the variability in radiation exposure across the samples. As with other indices, the range shows significant localized variation. The maximum value of 0.9 mSv/y represents a more significant contribution to total annual exposure in those specific hotspots, though it does not in itself imply an immediate health risk.

Overall, the data highlight areas of concern where radiological parameters exceed recommended safety limits, particularly in samples with high dose rates, Ra_e_q, and hazard indices. These findings underscore the importance of continued monitoring and mitigation efforts in regions with elevated radioactivity levels to minimize potential health risks to the population.

#### Correlation analysis

The radiological analysis of the studied monzogranite, as depicted by the correlation coefficient Pearson (Table S6), reveals significant relationships between elemental concentrations and radiation dose metrics. ^226^Ra exhibits exceptionally strong positive correlations (approaching or reaching 1) with key hazard indices, including Radium Equivalent Activity (Ra_e_q), External Hazard Index (Ex), Internal Hazard Index (In), Radioactive Level Index (Iɣ), Excess Lifetime Cancer Risk, and Annual Gonadal Equivalent Dose (AGED). This demonstrates that ^226^Ra is a dominant factor influencing these radiological hazard parameters and cancer risk assessments.

Similarly, Dose Rate, Outdoor Dose, and Indoor Dose show strong positive correlations with ^226^Ra, further emphasizing its central role in determining radiation exposure levels. ^238^U and ^40^ K exhibit moderate positive correlations with several dose metrics, including Dose Rate, Outdoor Dose, Indoor Dose, and Total Dose, indicating their notable contribution to overall radiation exposure. In contrast, ^232^Th displays only moderate positive correlations with Dose Rate, Outdoor Dose, and Indoor Dose, suggesting a comparatively lesser influence relative to ^238^U and ^40^ K. The strong inter correlations among dose metrics highlight their interdependence in evaluating radiation exposure. The high correlation coefficients between these parameters and Ra_e_q further underscore the critical role of radium content in determining radiation doses. In conclusion, the radiological assessment of El Fereyid monzogranite identifies ^226^Ra as a primary driver of radiation hazard indices and cancer risk, with significant implications for radiation protection and public health. The moderate correlations observed with^238^U and ^40^ K reflect the complex interplay of radionuclides in this geological setting, necessitating comprehensive monitoring and mitigation strategies to ensure public safety and effective radiation management.

**Fig. 6:**
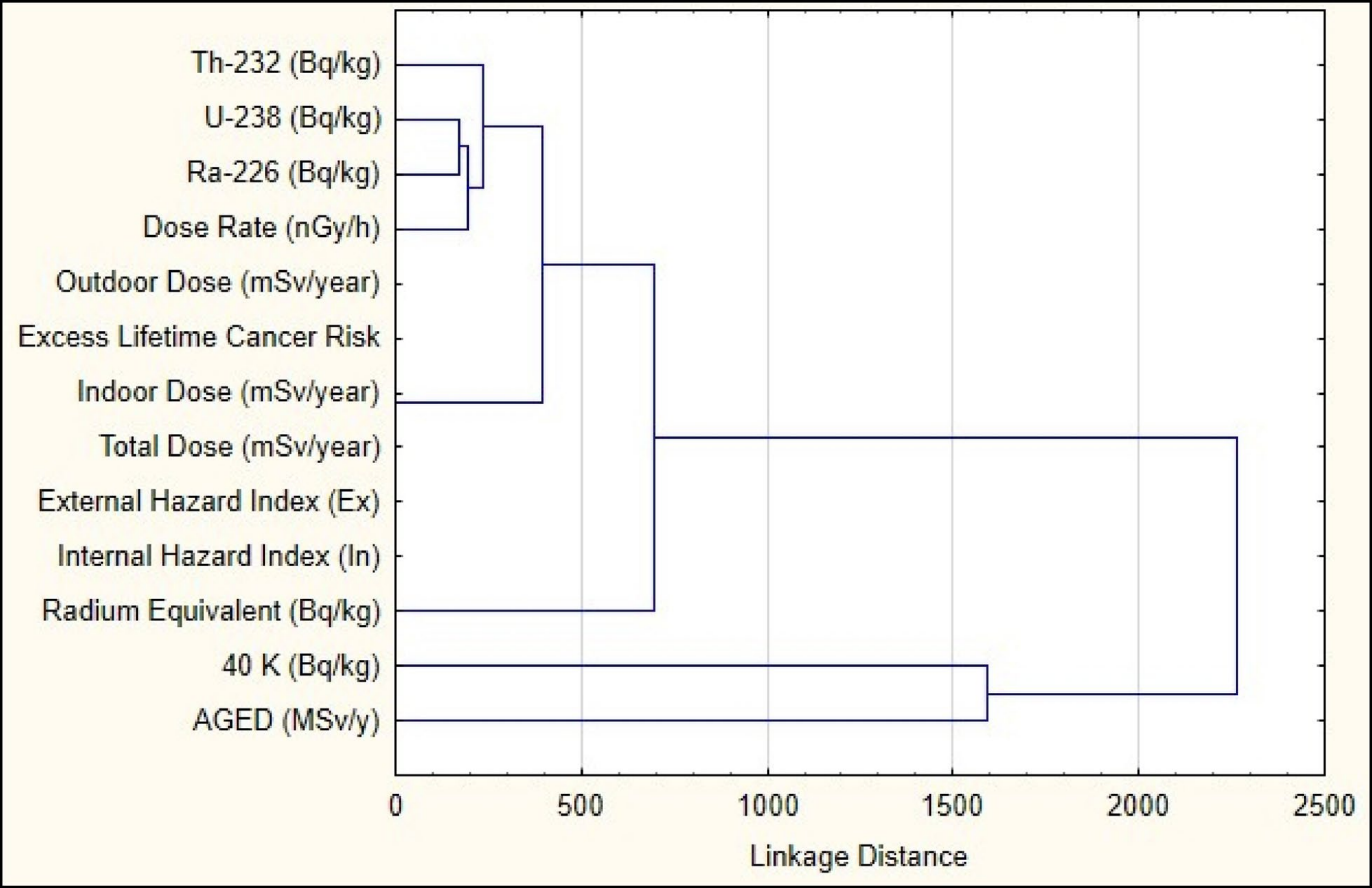
The clustering pattern demonstrates systematic relationships between the measured radiological variables in monzogranite of the study area.

The hierarchical cluster analysis (HCA) employing single linkage and Euclidean distances, as depicted in the dendrogram, reveals meaningful groupings among the radioactivity and radiation dose variables, underscoring their interrelationships in environmental and health risk assessments (Fig. 6). The early clustering of radionuclides such as ^232^Th, ^238^U , and ^226^Ra suggests a frequent geogenic origin or similar environmental behavior. Meanwhile, the close association between dose-related metrics (**Outdoor Dose**, **Indoor Dose**, and **Total Dose**) and hazard indices (**External Hazard Index**, **Internal Hazard Index**) highlights their shared dependence on radiation exposure pathways. This analysis not only validates the theoretical linkages between these variables but also provides a empirical basis for optimizing monitoring frameworks and risk assessment strategies in radiometric studies^8^. The presence of thorite, monazite, and zircon in the studied environment has significant implications for radiological hazards due to their inherent radioactive properties and geochemical behavior^44^. Thorite, a thorium-rich mineral, is a primary source of ^232^Th , which contributes to elevated radiation levels through its decay chain, including radium isotopes. This directly influences parameters such as Radium Equivalent Activity (Ra_e_q), external and internal hazard indices, and dose rates, as ^232^Th and its decay products emit both gamma radiation and alpha particles, posing risks from external exposure and inhalation of radon gas. Monazite, another thorium-bearing mineral, further amplifies radiological hazards due to its high concentrations of ^232^Th and ^238^U. The decay of these radionuclides in monazite contributes to increased Ra_e_q values and enhances gamma radiation levels, impacting outdoor and indoor dose rates.

Additionally, monazite’s resistance to weathering can lead to its accumulation in sediments, creating localized hotspots of radioactivity. Zircon, while primarily a zirconium silicate, often contains trace amounts of uranium and thorium as impurities. Although its contribution to overall radiation levels may be lower compared to thorite and monazite, zircon’s durability and widespread occurrence in geological formations make it a persistent source of low-level radiation, particularly in areas with high zircon concentrations^44^.

Collectively, the presence of thorite, monazite, and zircon underscores the importance of mineralogical composition in radiological hazard assessments. These minerals act as primary sources of ^232^Th and ^238^U, driving elevated radiation levels and influencing hazard indices such as Ra_e_q, external and internal hazard indices, and cancer risk metrics. Understanding the distribution and behavior of these minerals is crucial for identifying areas of heightened radiological risk, guiding effective monitoring and mitigation strategies, and ensuring public safety in regions with naturally occurring radioactive materials.

#### Comparative radiometric analysis

The activity concentrations for the El Fereyid monzogranite are presented in Table alongside data from key regional comparators and the world average^27,48–55^. The ^238^U and ^232^Th activities are moderately elevated above the global average for upper continental crust^27^yet are substantially lower than those characteristic of highly differentiated, uraniferous granites such as the El Nikeiba pluton in Egypt^48^ and suites fromTurkey^52^. Their values are most comparable to those reported from Jordan and Palestin^51,55^.

The most pronounced geochemical anomaly is the exceptionally low ^40^K activity (438.2 Bq/kg), which is profoundly depleted relative to all regional suites. It is less than half the values reported for Turkey, Iran, Spain^53^, and Jordan, and is negligible compared to the intensely potassic Hamra granite in Saudi Arabia (2846 Bq/kg)^49^ This is coupled with a high Th/U ratio (~ 1.5), indicating a relative enrichment of thorium or depletion of uranium when compared to the near-equilibrium conditions observed in many crustal rocks and the U-depleted signatures of granites in Spain and Iran^54^ (Table 2).

**Table 2:** Comparison of activity concentrations (Bq/kg) of natural radionuclides in the present study and previous studies.

Country/Region	^238^U(Bq/kg)	^232^Th (Bq/kg)	^40^ K (Bq/kg)	References
Egypt (El Fereyid area)	50	76.2	438.2	Present study
Egypt (El Nikeiba area)	153	171	1390	^48^
Saudi Arabia (Hamra)	89	77	2846	^49^
Egypt (General)	137	82	1082	^50^
Palestine	71	82	780	^51^
Turkey	80	101	974	^52^
Spain	84	42	1138	^53^
Iran	77	44.5	1017.2	^54^
Jordan	42	58	897	^55^
World average	33	45	412	^27^

This distinct geochemical fingerprint—showing moderate uranium and thorium levels alongside very low potassium and an unusual thorium-to-uranium ratio—points clearly to a history of intense fluid-driven alteration that occurred after the rock initially solidified. We propose that specific alteration processes, which transformed minerals into albite and sericite (fine-grained mica), effectively broke down the original potassium-rich feldspar. This explains the rock’s stark potassium deficiency. At the same time, thorium became relatively enriched because it tends to stay locked in place during these hydrothermal events, while uranium was partially dissolved and carried away by fluids due to its greater solubility, a process well-documented in similar rare-metal granite systems^56^.

## Conclusion remarks

This study establishes a direct petrogenetic link between accessory mineral chemistry and radiological hazards in the El Fereyid monzogranite. We demonstrate that thorite, monazite, and zircon are the primary hosts of Th and U, crystallized from a fractionated peraluminous melt. This specific mineral assemblage results in activity concentrations of ^232^Th and ^238^U that significantly exceed global averages. Statistical analyses confirm that Th-bearing minerals, particularly thorite, dominate the gamma radiation emissions. While the average radiological risk is low, we identify extreme, localized hotspots associated with thorite-rich zones that require careful management. This work provides a transferable model for assessing similar rare-metal granites globally, underscoring their dual nature as both critical metal resources and natural radiation sources.

## Supplementary Information

Below is the link to the electronic supplementary material.

Supplementary Material 1

## Data Availability

Data are contained within the article.
